# Ventilatory response to peripheral chemoreflex and muscle metaboreflex during static handgrip in healthy humans: evidence of hyperadditive integration

**DOI:** 10.1113/EP091094

**Published:** 2023-04-10

**Authors:** Diogo Machado de Oliveira, Thiago Ribeiro Lopes, Felipe Silva Gomes, Anas Rashid, Bruno Moreira Silva

**Affiliations:** ^1^ Graduate Program in Translational Medicine Federal University of São Paulo (Unifesp) São Paulo SP Brazil; ^2^ Paulista Association for the Development of Medicine (SPDM) São Paulo SP Brazil; ^3^ Department of Neuroscience ‘Rita Levi Montalcini’ University of Torino Torino Italy; ^4^ Graduate Program in Pulmonary Medicine Unifesp São Paulo SP Brazil; ^5^ Department of Physiology Unifesp São Paulo SP Brazil

**Keywords:** breathing, dyspnoea, isometric exercise, synergism

## Abstract

Exercise augments the hypoxia‐induced ventilatory response in an exercise intensity‐dependent manner. A mutual influence of hypoxia‐induced peripheral chemoreflex activation and exercise‐induced muscle metaboreflex activation might mediate the augmentation phenomenon. However, the nature of these reflexes' integration (i.e., hyperadditive, additive or hypoadditive) remains unclear, and the coactivation effect on breathing‐related sensations and emotions has not been explored. Accordingly, we investigated the effect of peripheral chemoreflex and muscle metaboreflex coactivation on ventilatory variables and breathing‐related sensations and emotions during exercise. Fourteen healthy adults performed 2‐min isocapnic static handgrip, first with the non‐dominant hand and immediately after with the dominant hand. During the dominant hand exercise, we (a) did not manipulate either reflex (control); (b) activated the peripheral chemoreflex by hypoxia; (c) activated the muscle metaboreflex in the non‐dominant arm by post‐exercise circulatory occlusion (PECO); or (d) coactivated both reflexes by simultaneous hypoxia and PECO use. Ventilation response to coactivation of reflexes (mean ± SD, 13 ± 6 l/min) was greater than the sum of responses to separated activations of reflexes (mean ± SD, 8 ± 8 l/min, *P* = 0.005). Breathing‐related sensory and emotional responses were similar between coactivation of reflexes and the sum of separate activations of reflexes. Thus, the peripheral chemoreflex and muscle metaboreflex integration during exercise appeared to be hyperadditive with regard to ventilation and additive with regard to breathing‐related sensations and emotions in healthy adults.

## Abstract (highlights)

- **What is the central question of this study?**
  What is the effect of peripheral chemoreflex and muscle metaboreflex integration on ventilation regulation, and what is the effect of integration on breathing‐related sensations and emotions?
- **What is the main finding and its importance?**
  Peripheral chemoreflex and muscle metaboreflex coactivation during isocapnic static handgrip exercise appeared to elicit a hyperadditive effect with regard to ventilation and an additive effect with regard to breathing‐related sensations and emotions. These findings reveal the nature of the integration between two neural mechanisms that operate during small‐muscle static exercise performed under hypoxia.

## 1 INTRODUCTION

Exercise augments hypoxia‐induced ventilatory response in an exercise intensity‐dependent manner (Weil et al., 1972). A mutual influence of hypoxia‐induced peripheral chemoreflex activation and exercise‐induced muscle metaboreflex activation might mediate the augmentation phenomenon (Fregosi & Seals, 1993). However, the nature of the integration of these reflexes remains unclear because some studies did not calculate the summed effect of reflexes activation (Gujic et al., 2007; Houssiere et al., 2005). Additionally, when it was calculated, coactivation and summed effects paradoxically produced similar ventilatory responses (Edgell & Stickland, 2014), representing an additive integration that supports no interaction between the reflexes (Wilson & Teppema, 2016). Two methodological aspects might have blunted the coactivation ventilatory response in a previous study (Edgell & Stickland, 2014), likely explaining the lack of interaction between the reflexes. First, the muscle metaboreflex was activated via post‐exercise circulatory occlusion (PECO) without contralateral limb exercise (Lam et al., 2019). Second, end‐tidal carbon dioxide partial pressure (end‐tidal $P_{{\mathrm{CO}}_{2}}$) was ∼7 mmHg lower during hypoxic PECO than during normoxic PECO (Alghaith et al., 2019). Moreover, the coactivation effect on breathing‐related sensations and emotions remains unexplored. Accordingly, we sought to test the hypothesis that peripheral chemoreflex and muscle metaboreflex coactivation during isocapnic exercise show hyperadditive integration, that is, they provoke greater ventilatory and breathing‐related sensation and emotional responses when activated together than the sum of effects yielded by each reflex activation separately.

## 2 METHODS

### 2.1 Ethical approval

All individuals signed a written informed consent before participating in the study. The study was conducted following the latest version of the *Declaration of Helsinki*, approved by the Ethics Committee of the Federal University of São Paulo (process: 1051/2017), and registered in the publicly available Plataforma Brasil database (CAEE: 74619517.5.0000.5505; National Council of Health, Ministry of Health, Brazil).

### 2.2 Participants

Seven men (age: 24.4 ± 3.6 years, body mass: 69.4 ± 8.8 kg, height: 1.72 ± 0.08 m) and seven women (age: 23.4 ± 3.15 years, body mass: 61.4 ± 9.5 kg, height: 1.66 ± 0.06 m) participated in the study. Nobody had previous exposure to acute or chronic hypoxia. Eligibility criteria were: age between 18 and 35 years, non‐smoker, body mass index <30 kg/m^2^, no diagnosis of chronic diseases, and being sedentary (i.e., not engaged in regular physical activities) or engaged in regular physical activities at most three times a week.

### 2.3 Experimental protocol

A first visit was used to familiarize the participants with the experimental procedures. Then, on two additional visits, we conducted experiments. The experimental visits occurred at the same period of the day, for a given participant, with an interval of at least 2 days and at most 7 days. Participants were instructed not to practice intense physical exercise for 48 h, not to ingest caffeine and alcohol for 24 h, and to ingest a light meal 2 h before the experiments. We sought to assess women during the beginning of the menstrual cycle, when oestrogen and progesterone levels are supposedly low, to increase the homogeneity of hormonal profiles. Ultimately, we managed to assess all women up to 8 days after the onset of menstruation.

### 2.4 Experimental procedures

**FIGURE 1:**
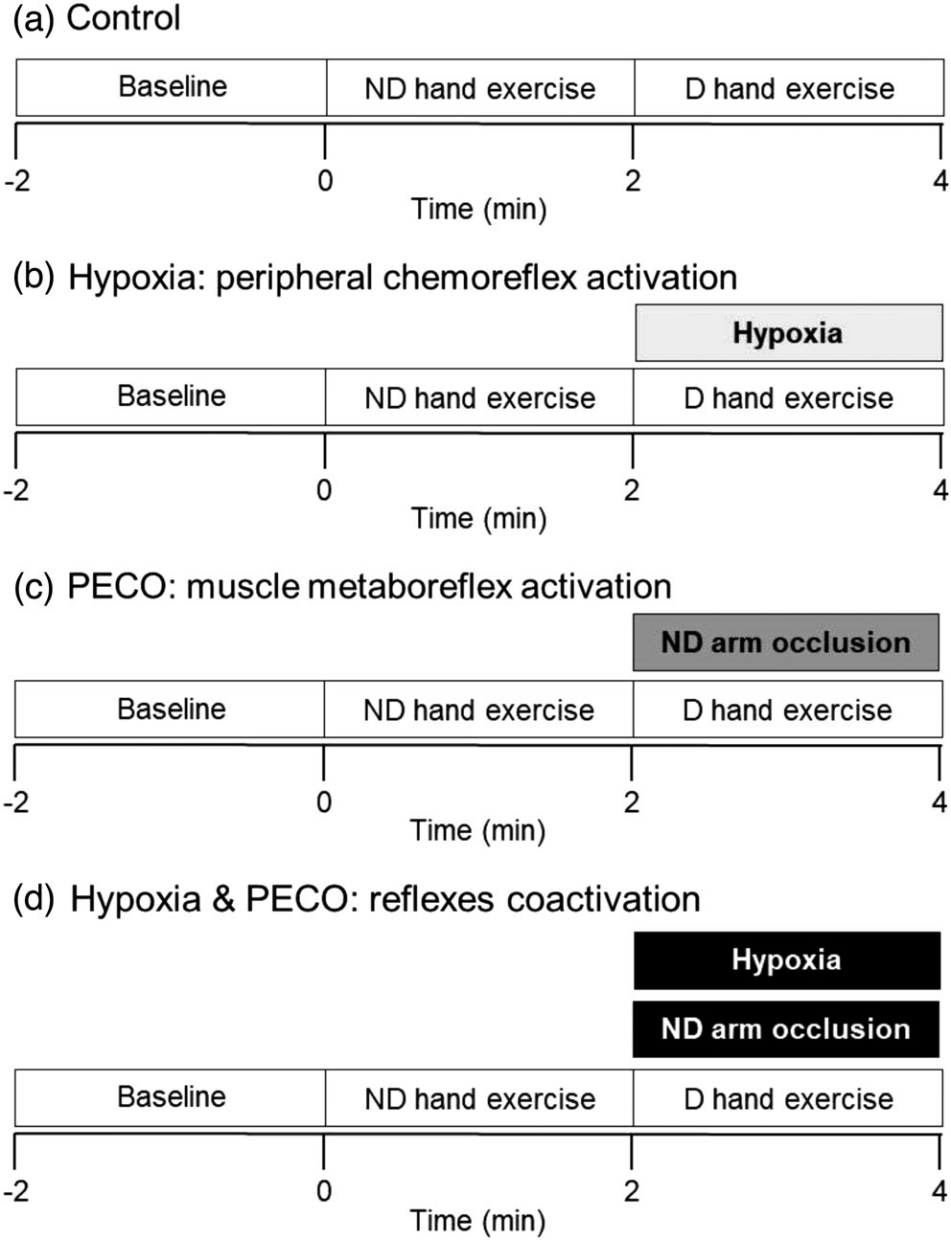
Experimental protocol. Participants performed 2‐min static handgrip under isocapnia, firstly with the non‐dominant hand and immediately after with the dominant hand. During the dominant hand exercise, we (a) did not manipulate both reflexes (control); (b) activated the peripheral chemoreflex by hypoxia; (c) activated the muscle metaboreflex in the non‐dominant arm by post‐exercise circulatory occlusion (PECO); or (d) coactivated both reflexes by simultaneous hypoxia and PECO use. D, dominant; ND, non‐dominant.

The participants were placed in the supine position on a large stretcher. The experiment had three 2‐min consecutive phases (Figure 1): (a) baseline resting, (b) static handgrip exercise with the non‐dominant hand, and (c) static handgrip exercise with the dominant hand. The experiment was repeated four times, varying the procedures during the dominant hand exercise. In this experimental phase, we (a) did not manipulate both reflexes (control); (b) activated the peripheral chemoreflex by hypoxia; (c) activated the muscle metaboreflex in the non‐dominant arm by PECO; or (d) coactivated both reflexes by simultaneous hypoxia and PECO use. Two experiments were conducted per day, in random order, with at least a 30 min interval between them. Participants were blinded to the concentration of O_2_ in the inspired air. Normoxia and hypoxia consisted of inhaling gas mixtures containing 21% O_2_ with 90% N_2_ and 10% O_2_ with 90% N_2_, respectively. Isocapnia was maintained throughout all experiments using a partial rebreathing set‐up (Silva et al., 2018).

The target handgrip load definition took into account a physiological criterion rather than a fixed percentage of maximal voluntary contraction because interindividual differences in physiological responses (Lee et al., 2021) and exercise tolerance (Hunter et al., 2009) can occur for a fixed percentage level. The target load was identified for each hand in the familiarization visit. The load had to provoke a progressive increase in perceived effort, attaining a perceived effort between 5 and 8 a.u. on a 0–10 Borg scale at the end of 2 min of exercise. This range of perceived effort is compatible with an exercise intensity enough to provoke accumulation of intramuscular metabolites without being so hard to accomplish (Lopes et al., 2022; West et al., 1995), in an attempt to avoid apnoeas and breathing pattern irregularities. Surface electromyogram (EMG) was later used to verify the presence of exercise‐induced metabolic stress, as the skeletal muscle's electrical activity increases during fatiguing static contractions (West et al., 1995). Muscle contraction metabolites were arrested by manually inflating a cuff placed on the non‐dominant arm to 220 mmHg before the end of the non‐dominant hand exercise.

### 2.5 Physiological measurements

An oro‐nasal silicon mask was tightly adjusted to the participants' faces. Airflow, O_2_ partial pressure ($P_{O_{2}}$) and CO_2_ partial pressure ($P_{{\mathrm{CO}}_{2}}$) were measured breath‐by‐breath (Quark CPET, Cosmed, Italy) by a bidirectional turbine, a paramagnetic sensor and a non‐dispersive infrared sensor, respectively. Then, pulmonary ventilation, breathing frequency, tidal volume, and end‐tidal $P_{O_{2}}$ and $P_{{\mathrm{CO}}_{2}}$ were calculated. Pulse oxygen saturation was measured by an oximetry sensor placed on a toe of the right foot (Nonin Medical, Plymouth, MN, USA). The measured values were time‐adjusted, considering a delay of 60 s between the monitoring in the foot and the oxyhaemoglobin desaturation in the central circulation during hypoxia exposure (Hamber et al., 1999). The electrical activity of the forearm flexor muscles was measured by two AgCl circular electrodes (MP43, MedPex, Republic of Korea) placed on the anteromedial surface of each forearm. A reference electrode was placed in the malleolus region of the right ankle. The raw EMG signal was sampled at 1 kHz, with a notch filter at 60 Hz (Dual Bioamp, ADInstruments, Bella Vista, NSW, Australia). The EMG signal was digitally filtered to a frequency bandwidth of 10−500 Hz (LabChart 3, ADInstruments). The root mean square (RMS) was calculated using 1‐s EMG data surrounding the peak force during a maximal voluntary contraction and 20‐s windows during the static handgrip exercise (LabChart 5, ADInstruments). Then, static handgrip exercise RMS data were converted into percentage values considering the maximal voluntary contraction RMS as 100% (West et al., 1995). Handgrip force was measured by a digital dynamometer (Grip Force Transducer, ADInstruments). The force tracing was projected on the ceiling, providing visual feedback to the participants.

### 2.6 Sensory and emotional measurements

Once at baseline and every minute during exercise, one of the researchers asked the following questions: ‘how intense is your sensation of breathing overall?’ (Lewthwaite & Jensen, 2021) and ‘how intense is your effort to perform the handgrip exercise?’ (Lopes et al., 2022). The first question sought to assess the general intensity of breathing sensation unidimensionally, considering that the awareness of breathing intensity starts earlier and is more intense than other qualitative descriptors during exercise in healthy adults (Lewthwaite & Jensen, 2021). The second question assessed the perceived effort to squeeze the hand dynamometer (Lopes et al., 2022). The participants had to verbally answer these two questions considering Borg's 0–10 scale, which was placed in their eyesight. During the exercise, we asked just two questions so as not to distract the participant in performing the handgrip task and to minimize the influence of speaking on ventilation. After each experiment, the participants sat on a chair to answer the Multidimensional Dyspnoea Profile (MDP) translated into Portuguese (Belo et al., 2019), considering their sensations and emotions at the end of the dominant hand exercise (i.e., focus period).

### 2.7 Analysis of reflexes integration

Ventilatory data corresponding to the baseline period and the last 20 s of the first and second minutes of the non‐dominant and dominant handgrip exercises were averaged. The last 20‐s ventilatory and subjective data of the dominant handgrip exercise during hypoxia, PECO and hypoxia combined with PECO were subtracted from the corresponding control data to calculate the effect of each experimental manipulation. According to a previous proposition (Wilson & Teppema, 2016), a coactivation effect greater than the sum of effects yielded by each reflex activation separately was considered a hyperadditive integration. Similar coactivation and summed effects denoted an additive integration. A coactivation effect smaller than the summed effect was considered an hypoadditive integration.

### 2.8 Statistical analyses

Data were analysed by either one‐way or two‐way repeated‐measures analysis of variance (RM ANOVA), followed by the Tukey HSD *post hoc* test, if needed. All analyses were done in the software Statistica (version 12, StatSoft, Tulsa, OK, USA). Results are reported as the mean and standard deviation. Statistical significance was interpreted as *P* < 0.05 in two‐tailed analyses.

## 3 RESULTS

The participants performed the handgrip exercise at 32 ± 6% of the maximal voluntary contraction force with the non‐dominant hand and 32 ± 5% with the dominant hand. The forearm muscles' EMG and perceived effort increased during the non‐dominant (EMG: 1st min: 35 ± 13 vs. 2nd min: 41% ± 14%, *P* = 0.001; effort: 1st min: 2.96 ± 1.0 vs. 2nd min: 5.36 ± 1.2 a.u., *P* < 0.001) and the dominant (EMG: 1st min: 34 ± 9 vs. 2nd min: 41% ± 8%, *P* = 0.001; effort: 1st min: 3.97 ± 1.0 vs. 2nd min: 6.24 ± 1.3 a.u., *P* = 0.001) handgrip exercise without differences among experiments (EMG: *P* = 0.140; effort: *P* = 0.187).

**FIGURE 2:**
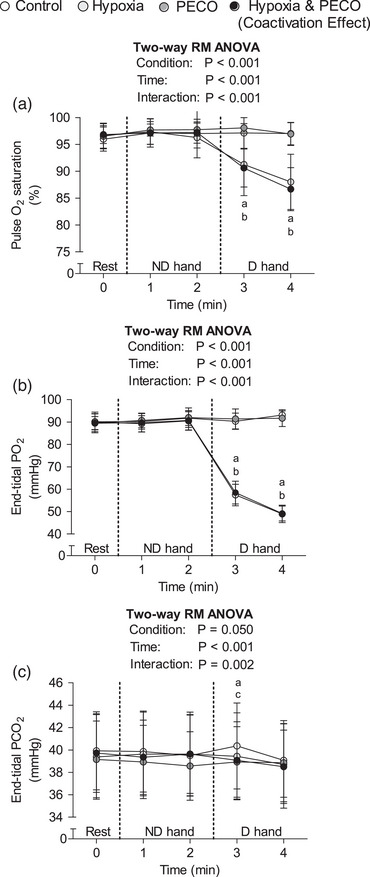
Time‐adjusted oxyhaemoglobin saturation (a), end‐tidal $P_{O_{2}}$ (b), and end‐tidal $P_{{\mathrm{CO}}_{2}}$ (c) at rest, 1st and 2nd min of non‐dominant hand exercise (minutes 1 and 2 on the abscissa), and 1st and 2nd min of dominant hand exercise (minutes 3 and 4 on the abscissa). Data presented as the mean and standard deviation (*n* = 14 for all variables) and analysed by two‐way repeated‐measures analysis of variance (RM ANOVA), followed by the Tukey *post hoc* test. (a) *P* < 0.05 hypoxia and PECO versus control; (b) *P* < 0.05 hypoxia versus control; (c) *P* < 0.05 PECO versus control. D, dominant; ND, non‐dominant; PECO, post‐exercise circulatory occlusion.

As expected, oxygen saturation and end‐tidal $P_{O_{2}}$ were lower in the hypoxia combined with PECO and in isolated hypoxia compared to the control during the dominant handgrip exercise (Figure 2a–c). In the first minute of the dominant handgrip exercise, end‐tidal $P_{{\mathrm{CO}}_{2}}$ was lower in the hypoxia combined with PECO and in the isolated PECO experiments than in the control experiment. In the second minute of the dominant handgrip exercise, end‐tidal $P_{{\mathrm{CO}}_{2}}$ was similar among all experiments.

**FIGURE 3:**
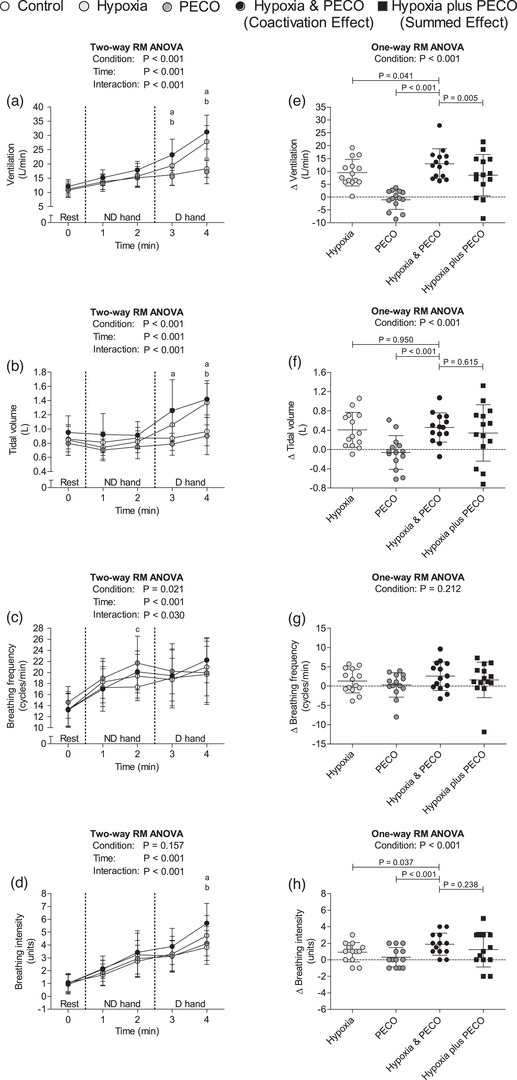
On the left, ventilation (a), tidal volume (b), breathing frequency (c), and breathing intensity (d) absolute values at rest, 1st and 2nd min of non‐dominant hand exercise (minutes 1 and 2 in the abscissa axis), and 1st and 2nd min of dominant hand exercise (minutes 3 and 4 in the abscissa axis). On the right, ventilation (e), tidal volume (f), breathing frequency (g), and breathing intensity (h) change versus control. Data presented as the mean and standard deviation (*n* = 14 for all variables) and analysed via two‐way (a–d) or one‐way (e–h) repeated measures analysis of variance (RM ANOVA), followed by the Tukey *post hoc* test. (a) *P* < 0.05 hypoxia and PECO versus control; (b) *P* < 0.05 hypoxia versus control; (c) *P* < 0.05 PECO versus control. D, dominant; ND, non‐dominant; PECO, post‐exercise circulatory occlusion.

During the dominant hand exercise, ventilation was higher in hypoxia combined with PECO and isolated hypoxia versus control (Figure 3a). In the first minute of this exercise, tidal volume was higher in hypoxia combined with PECO than in control (Figure 3b). Then, in the second minute, tidal volume was higher in hypoxia combined with PECO and in isolated hypoxia compared to control (Figure 3b). Breathing frequency was similar among experiments during the dominant hand exercise (Figure 3c). Breathing intensity was greater in hypoxia combined with PECO and in isolated hypoxia than in control in the second minute of the dominant hand exercise (Figure 3d).

Ventilation change was greater in hypoxia combined with PECO than in isolated hypoxia, isolated PECO and hypoxia plus PECO (i.e., hyperadditive integration; Figure 3e). Tidal volume change in hypoxia combined with PECO was greater than in isolated PECO, but similar to the changes in isolated hypoxia and hypoxia plus PECO (i.e., additive integration; Figure 3f). Breathing frequency changes were similar among experiments (i.e., additive integration; Figure 3g). Breathing intensity change was greater in hypoxia combined with PECO than in isolated hypoxia and in isolated PECO, but similar to the breathing intensity change in hypoxia plus PECO (i.e., additive integration; Figure 3h).

The intensity of respiratory muscle effort sensation was greater in hypoxia combined with PECO than in control (Table 1). The intensity of other breathing‐related sensations was similar among conditions. Hypoxia combined with PECO and hypoxia plus PECO changes were similar for all sensations obtained with the MDP (i.e., additive integration). The intensity of dyspnoea was greater in hypoxia combined with PECO than in control. Hypoxia combined with PECO and hypoxia plus PECO changes were similar for all emotions obtained with the MDP (i.e., additive integration).

**TABLE 1:** Multidimensional Dyspnoea Profile's sensory dimension, affective dimension 1 (dyspnoea) and 2 (other emotions) intensities.

	Control	Hypoxia	PECO	Hypoxia and PECO	*P*	Δ Hypoxia	Δ PECO	Δ Hypoxia and PECO	Δ Hypoxia plus PECO	*P*
**Sensory dimension**										
My breathing requires muscle work or effort (a.u.)	2.07 ± 2.27	2.86 ± 2.07	2.00 ± 2.04	3.64 ± 2.27^a^	0.001	0.79 ± 1.97	−0.07 ± 1.86	1.57 ± 1.87^b^	0.71 ± 3.69	0.021
I am not getting enough air or I feel hunger for air (a.u.)	1.14 ± 2.21	1.57 ± 2.03	1.36 ± 2.10	2.64 ± 2.92	0.064	0.43 ± 2.65	0.21 ± 0.80	1.50 ± 2.62	0.64 ± 3.13	0.112
My chest and lungs feel tight or constricted (a.u.)	0.29 ± 0.47	0.57 ± 1.16	0.29 ± 0.47	0.50 ± 0.85	0.567	0.29 ± 0.99	0.00 ± 0.68	0.21 ± 0.80	0.29 ± 1.33	0.701
My breathing requires mental effort or concentration (a.u.)	3.00 ± 2.51	3.93 ± 2.73	3.50 ± 2.77	4.00 ± 2.48	0.049	0.93 ± 1.49	0.50 ± 1.95	1.00 ± 1.66	1.43 ± 3.27	0.175
I am breathing a lot (a.u.)	1.85 ± 2.35	2.36 ± 2.71	2.14 ± 2.91	3.14 ± 3.57	0.151	0.50 ± 2.59	0.29 ± 1.54	1.29 ± 1.68	0.79 ± 3.64	0.468
**Affective dimension 1**										
Dyspnoea (a.u.)	2.21 ± 0.47	3.50 ± 0.54	2.85 ± 0.57	4.21 ± 0.53^a^	0.001	1.29 ± 1.33	0.64 ± 1.55	2.00 ± 1.66^b^	1.93 ± 2.67^c^	0.001
**Affective dimension 2**										
Depressed (a.u.)	0.07 ± 0.27	0.14 ± 0.53	0.07 ± 0.27	0.00 ± 0.00	—	0.07 ± 0.62	0.00 ± 0.39	−0.07 ± 0.27	0.07 ± 1.00	0.711
Anxious (a.u.)	2.00 ± 2.54	1.57 ± 0.94	1.36 ± 1.55	2.29 ± 2.33	0.598	−0.43 ± 2.53	−0.64 ± 3.56	0.29 ± 4.01	−1.07 ± 6.01	0.275
Frustrated (a.u.)	0.50 ± 1.87	0.21 ± 0.58	0.36 ± 0.93	0.14 ± 0.53	0.820	−0.29 ± 2.02	−0.14 ± 2.18	−0.36 ± 1.98	−0.43 ± 4.16	0.919
Angry (a.u.)	0.00 ± 0.00	0.14 ± 0.53	0.07 ± 0.27	0.00 ± 0.00	—	0.14 ± 0.53	0.07 ± 0.27	0.00 ± 0.00	0.21 ± 0.80	—
Afraid (a.u.)	0.43 ± 1.34	0.14 ± 0.36	0.07 ± 0.27	0.14 ± 0.36	0.527	−0.29 ± 1.14	−0.36 ± 1.39	0.00 ± 0.39	−0.64 ± 2.53	0.360

Data presented as means and standard deviation and compared among conditions with repeated measures ANOVA, followed by the Tukey *post hoc* test, when needed. *n* = 14. PECO, post‐exercise circulatory occlusion.

^a^ *P* < 0.05 hypoxia and PECO versus control.

^b^ *P* < 0.05 hypoxia and PECO versus PPECO.

^c^ *P* < 0.05 hypoxia plus PECO versus PECO.

## 4 DISCUSSION

The main result of the present study is that hypoxic air inhalation combined with metabolite arrest via PECO during exercise potentiated the ventilatory response versus isolated hypoxia and PECO exposure. As forearm EMG and perceived handgrip effort were similar between hypoxia and control during the dominant hand exercise, hypoxia likely increased ventilation via peripheral chemoreceptor activation (mainly the carotid chemoreceptors) rather than changing contracting muscle oxygenation and metabolism (Fregosi & Seals, 1993; Katayama et al., 2010). Furthermore, as both forearm EMG and perceived handgrip effort increased during exercise, exercise likely produced some metabolic stress resulting in metabolically sensitive muscle afferent activation during PECO (Bendahan et al., 1996; West et al., 1995). Therefore, the most likely explanation for the potentiated ventilatory response is an interaction between peripheral chemoreceptor and muscle metaboreceptor inputs.

Interestingly, PECO per se did not change ventilatory, sensory and emotional endpoints versus control. Perhaps, the dissimilar effect of PECO during normoxia and hypoxia is related to the amount of muscle metaboreceptors' afferent inputs. According to the study design, we limited the exercise intensity to avoid breathing irregularities and apnoeas. Then, perhaps, the level of afferent inputs from muscle metaboreceptors was not enough to augment ventilatory, sensory and emotional responses in PECO versus the control condition. This hypothesis is supported by the data reported in the study that advanced the PECO use on one limb while the other is exercising (Lam et al., 2019), because exercise intensity influenced the ventilation response.

The hyperadditive interaction is coherent with evidence that exercise augments hypoxia‐induced ventilatory responses in an exercise intensity‐dependent manner (Weil et al., 1972). Moreover, it supports the hypothesis that the integration of the reflexes had been underestimated before (Edgell & Stickland, 2014), likely due to the use of PECO at rest (Lam et al., 2019) and the lack of CO_2_ control (Alghaith et al., 2019). Our group previously reported that combined hypoxia‐induced peripheral chemoreflex activation and passive limb‐induced muscle mechanoreflex activation elicited a hyperadditive ventilatory response (Silva et al., 2018). Likewise, another group reported hypoxia‐induced peripheral chemoreflex activation and overall exercise‐induced muscle afferent activation elicited a hyperadditive ventilatory response (Wan et al., 2020). Therefore, present and past findings collectively support that both the mechanoreflex and the metaboreflex can potentiate the peripheral chemoreflex‐induced ventilatory response. The potentiation can be mediated within the central nervous system (Davies & Lahiri, 1973) or at the peripheral chemoreceptors (O'Regan, 1981), requiring further investigation. The lack of hyperadditive responses concerning breathing‐related sensations and emotions was unexpected and deserves investigation during whole‐body dynamic exercise, which presumably has a greater effect on these endpoints than small muscle static exercise (Lewthwaite & Jensen, 2021).

## 5 CONCLUSIONS

The peripheral chemoreflex and muscle metaboreflex integration appeared to be hyperadditive with regard to ventilation and additive with regard to breathing‐related sensations and emotions during isocapnic small‐muscle‐mass static exercise.

## AUTHOR CONTRIBUTIONS

Diogo Machado de Oliveira, Thiago Ribeiro Lopes, Bruno Moreira Silva: conception or design of the work. Diogo Machado de Oliveira, Thiago Ribeiro Lopes, Felipe Silva Gomes, Anas Rashid, Bruno Moreira Silva: acquisition, analysis, or interpretation of data for the work. Diogo Machado de Oliveira, Thiago Ribeiro Lopes, Felipe Silva Gomes, Anas Rashid, Bruno Moreira Silva: drafting of the work or revising it critically for important intellectual content. All authors have read and approved the final version of this manuscript and agree to be accountable for all aspects of the work in ensuring that questions related to the accuracy or integrity of any part of the work are appropriately investigated and resolved. All persons designated as authors qualify for authorship, and all those who qualify for authorship are listed.

## CONFLICT OF INTEREST

The authors have no conflict of interest to declare.

## Supporting information

Statistical Summary Document

## Data Availability

Data are available on request from the authors.
